# Disease-Induced Changes in Salivary Gland Function and the Composition of Saliva

**DOI:** 10.1177/00220345211004842

**Published:** 2021-04-17

**Authors:** G.B. Proctor, A.M. Shaalan

**Affiliations:** 1 Centre for Host Microbiome Interactions, Faculty of Dentistry, Oral & Craniofacial Sciences, King’s College London, London, UK

**Keywords:** salivation, oral health, mucins, Sjögren syndrome, secretion, sialadenitis

## Abstract

Although the physiological control of salivary secretion has been well studied, the impact of disease on salivary gland function and how this changes the composition and function of saliva is less well understood and is considered in this review. Secretion of saliva is dependent upon nerve-mediated stimuli, which activate glandular fluid and protein secretory mechanisms. The volume of saliva secreted by salivary glands depends upon the frequency and intensity of nerve-mediated stimuli, which increase dramatically with food intake and are subject to facilitatory or inhibitory influences within the central nervous system. Longer-term changes in saliva secretion have been found to occur in response to dietary change and aging, and these physiological influences can alter the composition and function of saliva in the mouth. Salivary gland dysfunction is associated with different diseases, including Sjögren syndrome, sialadenitis, and iatrogenic disease, due to radiotherapy and medications and is usually reported as a loss of secretory volume, which can range in severity. Defining salivary gland dysfunction by measuring salivary flow rates can be difficult since these vary widely in the healthy population. However, saliva can be sampled noninvasively and repeatedly, which facilitates longitudinal studies of subjects, providing a clearer picture of altered function. The application of omics technologies has revealed changes in saliva composition in many systemic diseases, offering disease biomarkers, but these compositional changes may not be related to salivary gland dysfunction. In Sjögren syndrome, there appears to be a change in the rheology of saliva due to altered mucin glycosylation. Analysis of glandular saliva in diseases or therapeutic interventions causing salivary gland inflammation frequently shows increased electrolyte concentrations and increased presence of innate immune proteins, most notably lactoferrin. Altering nerve-mediated signaling of salivary gland secretion contributes to medication-induced dysfunction and may also contribute to altered saliva composition in neurodegenerative disease.

## Introduction

Saliva is secreted by paired parotid, submandibular, and sublingual major salivary glands, along with hundreds of small, minor submucosal salivary glands. A further pair of “tubarial salivary glands” has very recently been identified and may provide a similar function in the oropharynx (Valstar et al. 2020). Oral health is dependent upon the continuous presence of saliva on the mucosal and tooth surfaces of the oral cavity, where it functions to maintain tooth mineralization and provide a protective, lubricating, renewing layer that interacts with and modifies the oral microbiota. Salivary glands must secrete sufficient volumes of saliva containing the components necessary for its rheological properties and other functions. Different diseases can cause salivary gland dysfunction, usually recorded as a reduced rate of salivary secretion. There have been fewer studies of the impact of disease on the composition of saliva. In this review, we consider the neural and cellular signaling that controls salivary secretion along with longer physiological influences in health, to provide background information before addressing how different diseases can affect salivary gland production and composition of saliva.

## Neural Regulation and Glandular Mechanisms of Salivary Secretion

**Figure 1.**
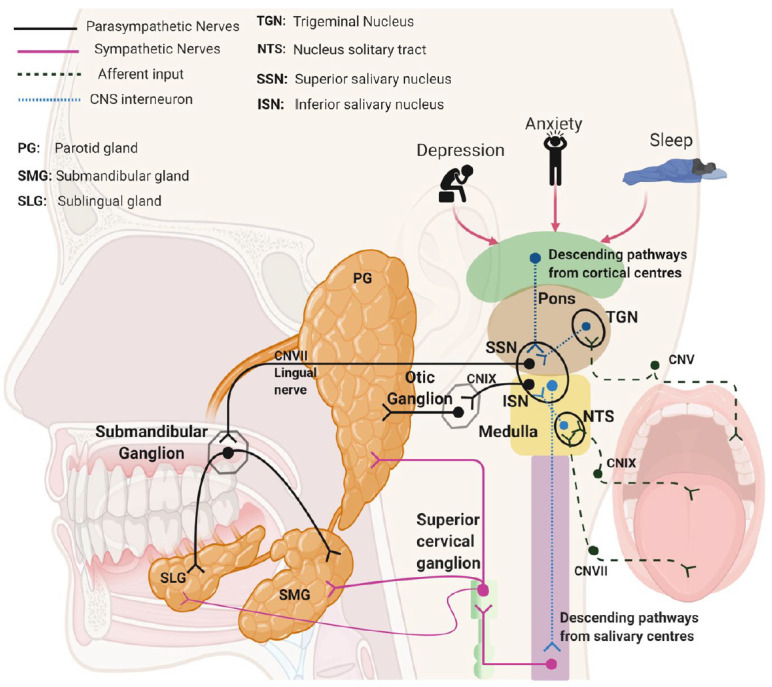
Nerve-regulated salivary secretion. Peripheral and central involvement in reflex-stimulated secretion by the major salivary glands.

**Figure 2.**
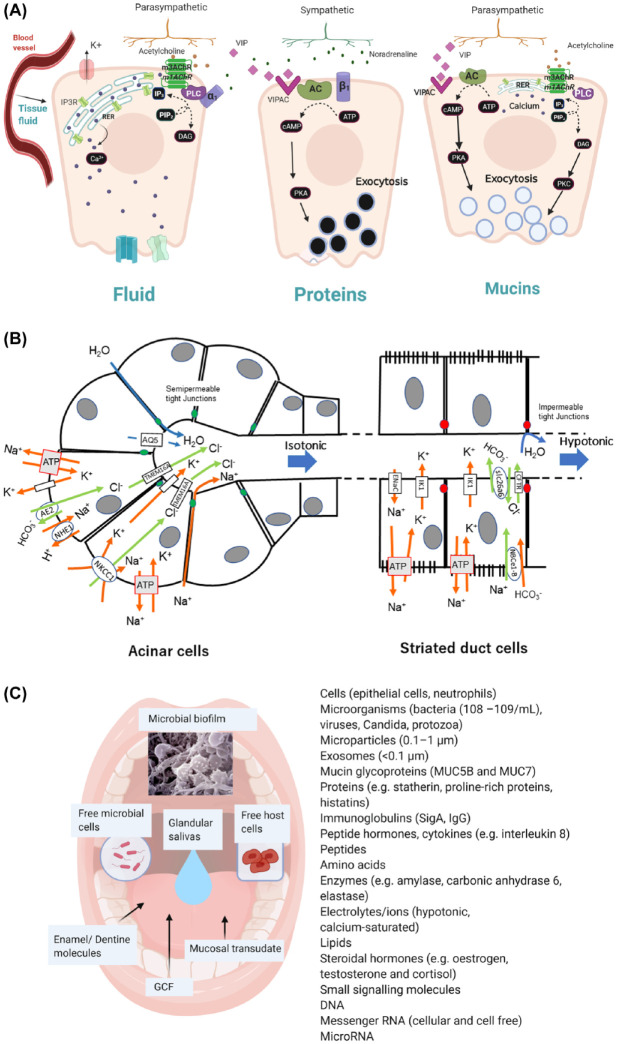
Salivary gland secretion of fluid and protein. (**A**) Coupling of nerve-mediated stimuli to secretion of fluid, proteins, and mucins by salivary acinar cells. Release of acetylcholine from parasympathetic (ps) nerves activates muscarinic receptors (m3/m1 AChRs), leading to activation of phospholipase C (PLC) and generation of IP_3_ (inositol triphosphate) from PIP_2_ (polyinositol biphosphate), binding to IP_3_ receptor and release of Ca^2+^ from RER (rough endoplasmic reticulum), increasing intracellular calcium concentration. Noradrenaline (NA) release from sympathetic (sy) nerves can cause a minor secretion of fluid through α1 adrenoceptors. Protein exocytosis is evoked by activation of PKA (protein kinase A) by intracellular cAMP generated from ATP by AC (adenylate cyclase) activated following NA binding to β1-adrenoceptors or vasointesinal polypeptide (VIP) signaling from ps nerves. Exocytosis from mucous acinar cells appears to occur without involvement of sy nerves. Acetylcholine release from ps nerves activates exocytosis through increased intracellular Ca^2+^ and PKC (protein kinase C) activated by DAG (diacylglycerol). VIP also signals mucous cell exocytosis through PKA activation (see Appendix). (**B**) Cellular mechanisms of fluid secretion. Acinar cell secretion of isotonic NaCl and water is followed by striated duct cell absorption of NaCl and secretion of KHCO_3_ in a hypotonic fluid. The principal membrane transporter proteins involved are shown. Acinar cell secretion is dependent on an apical chloride channel (TMEM16A), basolateral sodium, potassium cotransporter (NKCC1), antiports (AE2, NHE1), potassium channels, water channel (AQ5), and paracellular movement of sodium and water, all underpinned by sodium potassium ATPase. Ductal cell absorption of sodium and secretion of potassium through the ENaC and IK1 channels, respectively, is accompanied by secretion of bicarbonate through a combination of the CFTR chloride channel and the slc26a6 antiport, all ultimately dependent on sodium potassium ATPase (see Appendix). (**C**) Composition of saliva in the mouth. In addition to the saliva secreted by major and minor salivary glands, whole-mouth saliva contains components of non–salivary gland origin, including cells, bacteria and other microorganisms, cellular particles along with molecules contributed from gingival crevicular fluid (GCF), mucosal transudate, tooth enamel, and dentin.

The secretion of saliva is evoked by taste, mastication, and oral mechanoreception. Nerve-mediated signals are carried to the central nervous system by afferent sensory nerves, and efferent signals to salivary glands are carried in parasympathetic and sympathetic autonomic nerves (Proctor and Carpenter 2007). Parotid gland secretion is strongly influenced by mastication while different smells associated with food can evoke submandibular/sublingual gland secretion. Saliva is also secreted in response to trigeminal chemosensory stimulation of transient receptor potential (TRP) V1 and TRPM8 channels, by agonists such as capsaicin and menthol (Houghton et al. 2020). Central integration of sensory nerve signals in the salivary nuclei is subject to regulation by stimulatory or inhibitory signals descending from the cortical centers in the brain (Fig. 1). Muscarinic M3 receptors (m3AChRs), α_1_- and β1-adrenoceptors, and vasoactive intestinal polypeptide receptors play prominent roles in acinar cell stimulation–secretion coupling (Fig. 2A and Appendix). Acinar cell fluid secretion and ductal cell modification of saliva are driven by electrolyte concentration gradients created by active Na^+^, K^+^ ATPase transport in combination with a polarized distribution of cell membrane transport proteins (Fig. 2B and Appendix). The total protein concentration of saliva is mostly composed of proteins synthesized, stored, and exocytosed by acinar cells (see Appendix). Other non–salivary gland derived proteins in whole-mouth saliva are derived principally from gingival crevicular fluid. IgG is present in whole-mouth saliva at a concentration of approximately 0.016 mg/mL (1,000-fold lower than serum), while serum albumin is present at approximately 0.05 mg/mL (Gronblad 1982). There are other non–salivary gland derived components of whole-mouth saliva, including squamous epithelial cells, neutrophils, and microbial cells (Fig. 2C).

## Physiological Influences on Salivary Secretion and Composition

### Reflex-Stimulated and Resting Saliva

In the absence of stimulation provided by food consumption, salivary secretion is largely maintained at an “unstimulated” or “resting” flow rate. However, in conscious subjects, there is always some stimulation provided by the central nervous system. The “unstimulated” flow is approximately 0.3 to 0.4 mL/min but with wide variation between subjects and is sustained presumably by minor reflex activity and central nervous system activity since the flow of saliva decreases during sleep to around 0.1 mL/min. During food consumption, salivary secretion can increase over 5-fold compared to the unstimulated/resting flow rate.

The properties and composition of mixed saliva delivered to the mouth during eating differ from those in unstimulated saliva, reflecting altered contributions from each of the salivary glands. A greater proportion of stimulated saliva is of parotid origin, and since parotid saliva is a watery, non-mucin-containing secretion, the mixed saliva in the mouth is less viscoelastic. Viscoelastic mucin glycoproteins make a relatively greater contribution to whole-mouth saliva during rest (Veerman et al. 1996).

The nature of the reflex stimulus may also influence the composition of saliva. Changes in the proteome of whole-mouth saliva following trigeminal nerve–stimulating agonists have been revealed (Houghton et al. 2020). Bitter and other taste modalities and phenolic astringency have been found to cause acute changes in the salivary proteome (Neyraud et al. 2006).

The submandibular/sublingual, parotid, and labial minor submucosal glands contribute to a 12-h circadian rhythm of unstimulated saliva secretion, which shows a peak fluid flow in the mid-afternoon and a low point in the early morning (Dawes 1972; Wang et al. 2015). Time of day of saliva collection should therefore be considered when planning and executing studies on human subjects. Further considerations are the level of hydration and length of time following previous intake of food or drink since these also influence salivary flow rates (Dawes 1972).

### Autonomic Nerve–Mediated Trophism and Diet

Interruption of the autonomic nerve supply for extended periods causes salivary gland “disuse” atrophy in animal models and is more profound following parasympathectomy but also evident following sympathectomy (Proctor and Asking 1989). The trophic influence of nerves on human salivary glands was observed in submandibular glands transplanted to the lateral fornix for prevention of irreversible ocular dryness (Borrelli et al. 2010). Adherence to a liquid diet for 7 to 8 d decreased the volume and amylase content of parotid and whole-mouth saliva; the decrease was reversed on restoration of a solid diet (Hall et al. 1967). Whether increased reflex stimulation leads to salivary gland hypertrophy in humans is less certain. Reversible changes in parotid gland saliva composition, atrophy, and hypertrophy were observed in rats fed liquid and bulk diets, respectively (Johnson 1984; Takahashi et al. 2018). The submandibular and sublingual glands demonstrate little or no atrophy following a liquid diet (Scott and Gunn 1991; Takahashi et al. 2014).

### Aging and Cellular Senescence

Salivary gland histological structure changes with age, whereby the volume of epithelial secretory tissue is reduced and intraglandular fat and connective tissue increase (Scott 1977). Although previously disputed, a systematic review of the evidence indicates that salivary glands show age-dependent loss of function independent of polypharmacy (see later) manifested as a decrease in unstimulated but not stimulated whole-mouth salivary flow (Affoo et al. 2015). Studies in animal models have revealed age/senescence-dependent declines in salivary gland function (Miyagi et al. 2019).

There is modest evidence of altered saliva composition in elderly subjects. Concentrations of histatins in parotid and submandibular/sublingual saliva were found to be reduced in older age groups (Johnson et al. 2000). Analysis of saliva from self-reported disease-free elderly (60+ y old) subjects showed that salivary rheology (spinnbarkeit) and mucin (MUC7) content of unstimulated whole-mouth saliva were reduced compared with 18- to 30-y-old subjects (Pushpass et al. 2019). In a follow-up study, reduced MUC7 sialylation and mucoadhesion were detected, although symptoms of oral dryness were not reported. Altered submandibular gland mucin glycosylation with age has also been reported in the mouse (Kameyama et al. 2021).

## Disease-Induced Changes in Salivary Gland Function and the Composition of Saliva

**Figure 3.**
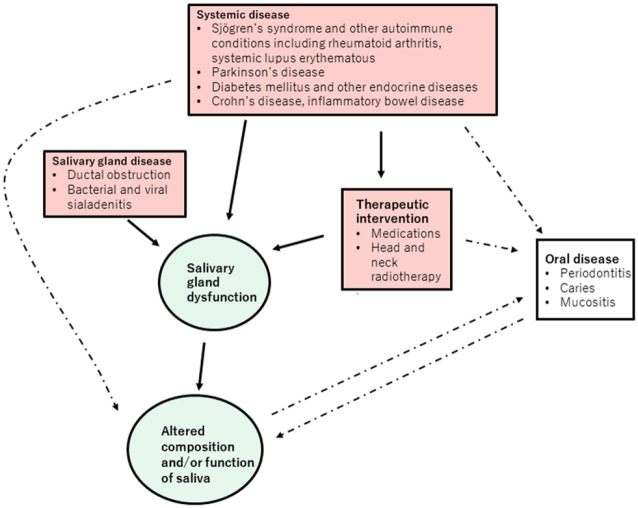
Relationships between disease, salivary gland dysfunction, and composition of saliva. The present review focuses on disease-related salivary gland dysfunction leading to altered composition and function of saliva. The causes considered are systemic diseases and therapeutic interventions (e.g., anticholinergic medications) that can cause salivary gland disease and dysfunction (solid lines). Saliva contains biomarkers of other systemic diseases (e.g., diabetes or liver disease) and oral diseases (e.g., periodontitis), which can directly alter the composition of saliva without necessarily causing salivary gland disease or dysfunction (dashed lines).

Salivary gland dysfunction can be described as the production of saliva, insufficient in terms of quantity and quality, as determined by composition, to preserve oral homeostasis and normal function. Different diseases and disease symptoms are linked with salivary gland dysfunction (Fig. 3). However, establishing salivary gland dysfunction can be problematic since there is a wide range of “normal” function. Measurement of whole-mouth salivary flow rates can reveal overall hypofunction (very low, less than 0.1 mL/min; low, 0.2 mL/min) while individual glands can be assessed by sampling ductal saliva. Salivary gland scintigraphy provides a simultaneous assessment of the function of each paired parotid and submandibular gland by quantitative imaging of the uptake and stimulated excretion of the radiotracer Tc-99m pertechnetate (Mandel and Mandel 2003).

Altered composition of whole-mouth saliva can reflect local, oral disease. The increased sensitivity of detection methods means that trace salivary components from the systemic circulation can be quantified, which has broadened the potential use of saliva as a diagnostic fluid. Altered composition of whole-mouth saliva or ductal saliva may therefore reflect more distant systemic diseases/pathologies without salivary gland dysfunction (Fig. 3). In this review, we focus on diseases that alter salivary gland secretion and saliva composition.

### Sjögren Syndrome

The impact of Sjögren syndrome (SjS) on salivary gland function has been extensively studied. A number of hypotheses have been put forward and evidence accumulated to explain the loss of function in salivary (and other exocrine) glands, and it may well be that a number of disease mechanisms contribute during the prolonged course of SjS (Mariette and Criswell 2018). Loss of secretory function has been attributed to infiltrating inflammatory cells, and although there may not be extensive tissue destruction early in the disease, it has been suggested that the inflammatory cytokines produced can influence acinar cell secretory function. The presence of focal infiltrates of inflammatory cells is a diagnostic feature of glandular involvement but appears not to be well correlated with loss of salivary secretory function. Studies in different animal models, including the nonobese diabetic (NOD) mouse and viral-mimicking Toll-like receptor 3 activation models, have confirmed that loss of secretory function may occur independently of infiltrating inflammatory cells (Jonsson et al. 2006; Bombardieri et al. 2012).

Salivary gland involvement is a component of the diagnostic criteria for SjS and is often confirmed by a labial gland biopsy. Measurement of salivary flow rates or assays of saliva components would allow repeated assessments of glandular function to monitor disease activity over time. However, whole-mouth salivary flow rate is not sufficiently specific to confirm salivary gland involvement in SjS. A large study of mixed groups of patients with salivary gland–related disease concluded that measurements of both submandibular/sublingual and parotid gland flow rates were required to provide sufficient specificity in confirming salivary gland involvement in primary SjS (Atkinson 1993). A combination of raised salivary sodium concentration and submandibular/sublingual saliva flow rate proved a useful biomarker (Pijpe et al. 2007). In comparison with healthy subjects and patients with non-SjS salivary gland disease, there were increased levels of soluble immune-related proteins, lactoferrin, β2-microglobulin, IgA, and autoantibodies in glandular and whole-mouth saliva from patients with SjS (Tabak et al. 1978; Carpenter et al. 2000; Katsiougiannis and Wong 2016). Early protein electrophoresis studies by Josie Beeley’s group demonstrated altered presence of unidentified proteins and were followed by the first use of a proteomics approach in which semiquantitative changes in a number of proteins were found, including lactoferrin, polymeric immunoglobulin receptor, and β2-microglobulin (Ryu et al. 2006). Following the advent of high-throughput proteomic analyses and characterization of the human salivary proteome, there have been a number of studies of the proteomics of saliva in SjS (Katsiougiannis and Wong 2016). The Wong laboratory has combined proteomics and transcriptomics approaches and identified multiple upregulated proteins and messenger RNAs (mRNAs) in whole-mouth saliva of patients with primary SjS compared with healthy control subjects; the proteins appeared to be mostly of non–salivary gland origin while the upregulated mRNAs suggested an interferon-inducible gene signature (Hu et al. 2007).

A proportion of patients with SjS complain of xerostomia even though their measured salivary flow rates do not confirm hypofunction, suggesting that the quality and composition of saliva might be changed (Osailan et al. 2011). The rheology of saliva is largely dependent on soluble mucins (MUC5B and MUC7) that contribute to the lubrication of oral surfaces. Compared with healthy control subjects, the extensional rheology of saliva from patients with SjS was reduced even in those without apparent glandular hypofunction. Mucin glycosylation but not concentration was reduced, which is likely to alter function (Chaudhury et al. 2016).

### Salivary Gland Obstructive Disease

Recurrent, chronic sialadenitis of the submandibular or parotid glands is most frequently associated with obstruction of the ductal system due to stones and/or ductal stricture with episodes of microbial infection associated with salivary stasis in the ductal system (Iro et al. 2009; Zhang et al. 2019). Clinical investigation is directed at establishing the presence of ductal obstruction using sialography, and the dysfunction relates to swelling and pain associated with “meal-time” syndrome. Minimally invasive salivary gland surgery and lithotripsy are now used to treat obstructive disease, and a few studies have assessed secretory dysfunction. Parotid secretory function was improved following removal of the ductal obstruction, as assessed by scintigraphy (99mTc pertechnetate) and sialometry (Zhang et al. 2019).

Studies on animal models have demonstrated that ductal ligation–induced glandular obstruction causes a rapid loss of secretory function that is not dependent on inflammatory cell infiltration of glands (Correia et al. 2008). Saliva chloride and sodium concentrations were increased, suggesting that ductal cell function is also affected. Even following prolonged periods of obstruction and extreme glandular atrophy, functional recovery can take place with secretion of saliva containing a normal protein composition (Osailan et al. 2006).

### Salivary Gland Infection

A longitudinal study of acute bacterial sialadenitis in a patient with chronic recurrent parotitis demonstrated rapid loss of secretory function accompanied by increased presence of innate immune proteins (lactoferrin and lysozyme), immunoglobulins, and albumin, which resolved over a 2- to 3-wk period, although secretory function remained lower than the control gland (Tabak et al. 1978). Several viruses are associated with salivary gland inflammation, including paramyxovirus (mumps), cytomegalovirus, hepatitis C, human immunodeficiency virus (HIV), and human herpes virus. The secretion and composition of submandibular/sublingual (SMSL) saliva in HIV-infected patients was assessed (Jainkittivong et al. 2009). Unstimulated and citric acid–stimulated SMSL saliva secretion was reduced in highly active antiretroviral therapy (HAART)–treated and non-HAART patient groups. Saliva contained increased concentrations of the mucins MUC5B and MUC7, which have an antiviral activity, but secretory output of both mucins over time was similar in HIV-positive and control subjects. Lower SMSL saliva flow rates and blood CD4^+^ counts were seen in *Candida*-positive compared with *Candida*-negative HIV-positive subjects, but there was no association between *Candida* and salivary mucin concentrations. Influenza A (H3N2) is associated with parotitis, and more recently, the presence of severe acute respiratory syndrome coronavirus 1 (SARS-CoV-1) in salivary glands and association of severe acute respiratory syndrome coronavirus 2 (SARS-CoV-2) with saliva have been described, but it is unclear whether these viruses affect salivary gland function (Chern et al. 2020).

### Medication-Induced Salivary Gland Dysfunction

Medication and salivary dysfunction were considered in a series of systematic reviews that emerged from the World Workshop on Oral Medicine VI (Wolff et al. 2017). It is clear from these reviews that many medications cause xerostomia as a side effect, but very few have been tested for objective changes in salivary flow. The principal peripheral target of medications causing salivary gland dysfunction is the m3AChR and, to a lesser extent, m1AChR of salivary gland acinar cells, which are blocked by antimuscarinic drugs used in the treatment of irritable bladder and chronic obstructive pulmonary disease. Antihypertensive α2-adrenoceptor agonists and antidepressants that inhibit noradrenaline and serotonin reuptake appear to act centrally by inhibiting nerve-mediated transmission in descending pathways acting on the salivary centers (Moreira Tdos et al. 2002). There have been few studies of the composition of saliva in relation to medication-induced salivary gland dysfunction. Nonselective and β1-adrenergic receptor antagonists in humans altered the composition of saliva by reducing the amount of total protein, histatin, statherin, and amylase (Jensen et al. 1994). Similar effects were also shown with nonselective (propranolol) and the β1-selective (atenolol) adrenergic receptor antagonists (Nederfors 1996). More studies are needed to identify the effects of new medications on composition and flow rate of saliva from both minor and major salivary glands. Acute dosage with β1-selective or nonselective β-adrenoceptor antagonists reduces total salivary protein secretion, and longer-term application alters salivary protein expression in studies on animal models (Johnson and Cortez 1988).

### Irradiation—External Beam

Xerostomia after radiotherapy (RT) for head and neck cancer (HNC) is a very frequent complication that has a major impact on quality of life in HNC survivors. Loss of secretory function in coirradiated salivary glands occurs early following treatment, even before apparent loss of secretory acinar cells, but progresses to approximately 10% of control levels of salivation as secretory cells die and are not replaced (Vissink et al. 2010). Valdez et al. (1992) compared parotid and submandibular/sublingual gland function approximately 2 y following irradiation therapy and found similar levels of irradiation dose-dependent reduction, compared with unirradiated control subjects. Sialochemistry revealed reduced total protein secretion but increased secretion of lactoferrin. Ductal dysfunction was indicated by increased concentrations of chloride and sodium even though salivary flow rates were reduced.

The severity of xerostomia following therapeutic irradiation can be reduced by using parotid gland–sparing intensity-modulated radiotherapy (IMRT) in treating patients for HNC. In a longitudinal study, at 3 to 6 mo after IMRT, stimulated and unstimulated parotid saliva flow rates were significantly decreased compared with baseline before IMRT but showed some recovery by 12 mo (Richards et al. 2017). Compositional analysis revealed reduced total protein secretion that recovered at 12 mo while increased lactoferrin concentration persisted. Overall, more severe xerostomia was associated with lower parotid saliva flow rates.

### Radioactive Iodine and Radiolabeled Prostate-Specific Membrane Antigen Infusions

A substantial proportion of thyroid cancer sufferers are treated with radioactive iodine (RAI), which is also concentrated in parotid and submandibular glands by expressed sodium iodide membrane symporter proteins. Acute salivary gland hypofunction is seen in the first week following treatment and chronic sialadenitis from 3 mo onward, associated with ductal strictures and obstructive disease, which causes pain and associated salivary gland dysfunction, usually determined by salivary gland scintigraphy (Singer et al. 2020).

Radioactive actinium (225Ac)– or lutetium (177Lu)–labeled prostate-specific membrane antigen (PSMA)–617 are low molecular weight ligands of prostate-specific membrane antigen and are used in the treatment of prostate cancer. Dry mouth is the main side effect of this therapy owing to uptake of radioligand by PSMA expressing salivary glands causing salivary gland hypofunction as determined by salivary gland scintigraphy (Rathke et al. 2018). The impact of treatment on salivary volume and composition has not been determined.

### Hematopoietic Stem Cell Transplantation

Autologous or allogeneic hematopoietic stem cell transplantation (HSCT) is given for the treatment of leukemia, malignant lymphoma, and other blood malignancies and is preceded by a conditioning chemotherapy and/or radiotherapy. An early complication of treatment is oral mucositis due to the conditioning therapy while graft-versus-host reactions may occur acutely or chronically. Salivary flow rates are reduced following HSCT, but the reported time span differs between published studies.

A recent review of published studies showed a trend toward decreasing salivary flow rate days and months after HSCT and reported compositional changes in saliva reflecting an inflammatory response directly after HSCT, followed by increases in salivary antimicrobial defense mechanisms at 6 mo after HSCT (van Leeuwen et al. 2019). In a longitudinal study, there was an early reduction in salivary fluid secretion but not total protein secretion (van Leeuwen et al. 2018). An early phase of oral mucosal–associated pathology coincided with increased salivary albumin. During this period, numbers of circulating neutrophils were decreased, and salivary levels of the antimicrobial proteins, including IgA, myeloperoxidase, and neutrophil-derived α-defensin, were decreased. In this period, there appeared to be salivary gland inflammation since salivary lactoferrin levels were elevated, suggesting that glandular expression of this antimicrobial protein might be compensating for loss of other oral antimicrobial activity. Previous studies have similarly shown decreases in IgA and increases in lactoferrin, secretory leukocyte protease inhibitor, and β2-microglobulin after HSCT (Imanguli et al. 2007) .

### Neurodegenerative Disease

Autonomic dysfunction involving the gastrointestinal system is a frequent early manifestation of Parkinson disease (PD). Sialorrhea is problematic in PD, but the drooling and swallowing difficulties experienced by sufferers are due to loss of muscular control rather than hypersalivation. To reduce drooling and inhalation of saliva, botulinum toxin is sometimes injected into major salivary glands (Ruiz-Roca et al. 2019). Intraneuronal inclusions of aggregated α-synuclein protein, termed *Lewy pathology*, are the pathological hallmark of PD and have been found in the submandibular glands as well as in multiple sites of the autonomic nervous system involved in control of saliva production in patients with PD (Beach et al. 2010). Paradoxically, a number of studies have demonstrated decreased saliva secretion in PD. An assessment of salivary secretion in subjects with PD, “on” or “off” levodopa therapy, demonstrated reduced unstimulated salivary flow rates compared with non-PD control subjects; salivary flow was greater in PD levodopa-treated compared to untreated PD subjects. The latter effect appeared to be exerted through a central mechanism since treatment with the peripheral dopaminergic D_2_ receptor antagonist domperidone did not reduce salivary flow (Tumilasci et al. 2006).

A further study of salivary flow and protein composition in PD patients compared with age-matched control subjects found similar salivary flow rates in both groups, but total protein, amylase, and albumin concentrations were higher in the PD group, whether on or off levodopa, suggesting altered glandular function and oral epithelial integrity (Masters et al. 2015).

In Alzheimer disease (AD), submandibular but not parotid gland secretion is reduced (Ship et al. 1990). A recent study found reduced lactoferrin concentration in unstimulated whole-mouth saliva to be specific to AD, which suggests a deficit in salivary gland expression or secretion of lactoferrin, but the disease mechanism linking salivary lactoferrin and AD is at present unclear (Gonzalez-Sanchez et al. 2020).

### Bulimia Nervosa

Parotid gland enlargement has been varyingly reported in bulimia nervosa. An early study reported enlargement in 25% of patients with binge eating syndrome of bulimia nervosa, and it appeared to be correlated with raised parotid salivary amylase and total protein concentrations and increased serum levels of salivary type amylase (Kronvall et al. 1992). Dynesen et al. (2008) found reduced unstimulated whole-mouth salivary flow in bulimia nervosa patients coinciding with increased xerostomia, primarily due to the intake of antidepressant medications. Nevertheless, following adjustment for medication intake, there was a residual effect of bulimia nervosa reducing unstimulated whole-mouth salivary secretion.

### Crohn Disease

The incidence and severity of xerostomia have been found to be correlated with the severity of Crohn disease (CD) (de Vries et al. 2018). Reduced unstimulated salivary flow was found in a recent study of CD subjects, but further studies are required to determine whether altered salivary gland function is responsible for the recorded xerostomia. Raised secretory immunoglobulin A (SIgA) and IgA levels in saliva have been detected in CD alone and in combination with orofacial granulomatosis, an associated oral inflammatory phenotype, which is also characterized by raised concentrations of lactoferrin (Janšáková et al. 2021). The raised SIgA and lactoferrin concentrations suggest altered salivary gland function or inflammation, possibly in major or minor salivary glands.

## Conclusion

Salivary gland function is dependent on nerve-mediated stimuli, which exert acute control of saliva secretion and can alter function through longer-term trophic influences. A reduction in nerve-mediated stimuli may be a contributory factor in addition to iatrogenic reductions in salivary flow rates due to medications taken by elderly subjects. Salivary gland dysfunction manifested as reductions in salivary flow rate is observed in different diseases and often accompanied by the perception of dry mouth, particularly where there is widespread involvement of salivary glands as in advanced Sjögren syndrome and following therapeutic irradiation. Relatively few studies have addressed how salivary gland dysfunction alters the composition and function of secreted saliva and the mechanisms underlying such disease-induced changes.

## Author Contributions

G.B. Proctor, contributed to conception, design, and data interpretation, drafted and critically revised the manuscript; A.M. Shaalan, contributed to design and data interpretation, drafted and critically revised the manuscript. Both authors gave final approval and agree to be accountable for all aspects of the work.

## Supplemental Material

sj-pdf-1-jdr-10.1177_00220345211004842 – Supplemental material for Disease-Induced Changes in Salivary Gland Function and the Composition of Saliva Supplemental material, sj-pdf-1-jdr-10.1177_00220345211004842 for Disease-Induced Changes in Salivary Gland Function and the Composition of Saliva by G.B. Proctor and A.M. Shaalan in Journal of Dental Research

## References

[bibr1-00220345211004842] Affoo RH Foley N Garrick R Siqueira WL Martin RE . 2015. Meta-analysis of salivary flow rates in young and older adults. J Am Geriatr Soc. 63(10):2142–2151. 26456531 10.1111/jgs.13652

[bibr2-00220345211004842] Atkinson JC . 1993. The role of salivary measurements in the diagnosis of salivary autoimmune-diseases. Ann N Y Acad Sci. 694:238–251. 8215059 10.1111/j.1749-6632.1993.tb18357.x

[bibr3-00220345211004842] Beach TG Adler CH Sue LI Vedders L Lue L White CL Akiyama H Caviness JN Shill HA Sabbagh MN , et al. 2010. Multi-organ distribution of phosphorylated alpha-synuclein histopathology in subjects with Lewy body disorders. Acta Neuropathol. 119(6):689–702. 20306269 10.1007/s00401-010-0664-3 PMC2866090

[bibr4-00220345211004842] Bombardieri M Barone F Lucchesi D Nayar S van den Berg WB Proctor G Buckley CD Pitzalis C. 2012. Inducible tertiary lymphoid structures, autoimmunity, and exocrine dysfunction in a novel model of salivary gland inflammation in C57BL/6 mice. J Immunol. 189(7):3767–3776. 22942425 10.4049/jimmunol.1201216 PMC3448973

[bibr5-00220345211004842] Borrelli M Schroder C Dart JK Collin JR Sieg P Cree IA Matheson MA Tiffany JM Proctor G van Best J , et al. 2010. Long-term follow-up after submandibular gland transplantation in severe dry eyes secondary to cicatrizing conjunctivitis. Am J Ophthalmol. 150(6):894–904. 20920813 10.1016/j.ajo.2010.05.010

[bibr6-00220345211004842] Carpenter GH Proctor GB Pankhurst CL O’Donohue J Scott D Hunnable MP . 2000. Sialochemical markers of salivary gland involvement with Sjogren’s syndrome secondary to rheumatoid arthritis and primary biliary cirrhosis. J Oral Pathol Med. 29(9):452–459. 11016688 10.1034/j.1600-0714.2000.290906.x

[bibr7-00220345211004842] Chaudhury NMA Proctor GB Karlsson NG Carpenter GH Flowers SA . 2016. Reduced mucin-7 (Muc7) sialylation and altered saliva rheology in Sjogren’s syndrome associated oral dryness. Mol Cell Proteomics. 15(3):1048–1059. 26631508 10.1074/mcp.M115.052993 PMC4813687

[bibr8-00220345211004842] Chern A Famuyide AO Moonis G Lalwani AK . 2020. Sialadenitis: a possible early manifestation of COVID-19. Laryngoscope. 130(11):2595–2597. 10.1002/lary.29083 PMC7461412 32833242

[bibr9-00220345211004842] Correia PN Carpenter GH Osailan SM Paterson KL Proctor GB . 2008. Acute salivary gland hypofunction in the duct ligation model in the absence of inflammation. Oral Dis. 14(6):520–528. 18221457 10.1111/j.1601-0825.2007.01413.x PMC2592348

[bibr10-00220345211004842] Dawes C . 1972. Circadian rhythms in human salivary flow rate and composition. J Physiol. 220(3):529–545. 5016036 10.1113/jphysiol.1972.sp009721 PMC1331668

[bibr11-00220345211004842] de Vries SAG Tan CXW Bouma G Forouzanfar T Brand HS de Boer NK . 2018. Salivary function and oral health problems in Crohn’s disease patients. Inflamm Bowel Dis. 24(6):1361–1367. 29718221 10.1093/ibd/izy017

[bibr12-00220345211004842] Dynesen AW Bardow A Petersson B Nielsen LR Nauntofte B . 2008. Salivary changes and dental erosion in bulimia nervosa. Oral Surg Oral Med Oral Pathol Oral Radiol Endod. 106(5):696–707. 18805715 10.1016/j.tripleo.2008.07.003

[bibr13-00220345211004842] Gonzalez-Sanchez M Bartolome F Antequera D Puertas-Martin V Gonzalez P Gomez-Grande A Llamas-Velasco S Herrero-San Martin A Perez-Martinez D Villarejo-Galende A , et al. 2020. Decreased salivary lactoferrin levels are specific to Alzheimer’s disease. Ebiomedicine. 57:102834. 32586758 10.1016/j.ebiom.2020.102834 PMC7378957

[bibr14-00220345211004842] Gronblad EA . 1982. Concentration of immunoglobulins in human whole saliva—effect of physiological stimulation. Acta Odontol Scand. 40(2):87–95. 6954831 10.3109/00016358209041120

[bibr15-00220345211004842] Hall HD Merig JJ Jr Schneyer CA . 1967. Metrecal-induced changes in human saliva. Proc Soc Exp Biol Med. 124(2):532–536. 6019888 10.3181/00379727-124-31781

[bibr16-00220345211004842] Houghton JW Carpenter G Hans J Pesaro M Lynham S Proctor G. 2020. Agonists of orally expressed TRP channels stimulate salivary secretion and modify the salivary proteome. Mol Cell Proteomics. 19(10):1664–1676. 32651226 10.1074/mcp.RA120.002174 PMC8014997

[bibr17-00220345211004842] Hu S Wang JH Meijer J Leong S Xie YM Yu TW Zhou H Henry S Vissink A Pijpe J , et al. 2007. Salivary proteomic and genomic biomarkers for primary Sjogren’s syndrome. Arthritis Rheum. 56(11):3588–3600. 17968930 10.1002/art.22954 PMC2856841

[bibr18-00220345211004842] Imanguli MM Atkinson JC Harvey KE Hoehn GT Ryu OH Wu TX Kingman A Barrett AJ Bishop MR Childs RW , et al. 2007. Changes in salivary proteome following allogeneic hematopoietic stem cell transplantation. Exp Hematol. 35(2):184–192. 17258067 10.1016/j.exphem.2006.10.009 PMC1832107

[bibr19-00220345211004842] Iro H Zenk J Escudier MP Nahlieli O Capaccio P Katz P Brown J McGurk M. 2009. Outcome of minimally invasive management of salivary calculi in 4,691 patients. Laryngoscope. 119(2):263–268. 19160432 10.1002/lary.20008

[bibr20-00220345211004842] Jainkittivong A Lin AL Johnson DA Langlais RP Yeh CK . 2009. Salivary secretion, mucin concentrations and Candida carriage in HIV-infected patients. Oral Dis. 15(3):229–234. 19207880 10.1111/j.1601-0825.2009.01514.x

[bibr21-00220345211004842] Janšáková K Escudier M Tóthová Ľ Proctor G. 2021. Salivary changes in oxidative stress related to inflammation in oral and gastrointestinal diseases. Oral Dis. 27(2):280–289. 32643850 10.1111/odi.13537

[bibr22-00220345211004842] Jensen JL Xu T Lamkin MS Brodin P Aars H Berg T Oppenheim EG . 1994. Physiological regulation of the secretion of histatins and statherins in human parotid saliva. J Dent Res. 73(12):1811–1817. 7814752 10.1177/00220345940730120401

[bibr23-00220345211004842] Johnson DA . 1984. Changes in rat parotid salivary proteins associated with liquid diet-induced gland atrophy and isoproterenol-induced gland enlargement. Arch Oral Biol. 29(3):215–221. 6587843 10.1016/0003-9969(84)90058-x

[bibr24-00220345211004842] Johnson DA Cortez JE . 1988. Chronic treatment with beta-adrenergic agonists and antagonists alters the composition of proteins in rat parotid-saliva. J Dent Res. 67(8):1103–1108. 2900257 10.1177/00220345880670080801

[bibr25-00220345211004842] Johnson DA Yeh CK Dodds MWJ . 2000. Effect of donor age on the concentrations of histatins in human parotid and submandibular/sublingual saliva. Arch Oral Biol. 45(9):731–740. 10869486 10.1016/s0003-9969(00)00047-9

[bibr26-00220345211004842] Jonsson MV Delaleu N Brokstad KA Berggreen E Skarstein K. 2006. Impaired salivary gland function in NOD mice—association with changes in cytokine profile but not with histopathologic changes in the salivary gland. Arthritis Rheum. 54(7):2300–2305. 16802370 10.1002/art.21945

[bibr27-00220345211004842] Kameyama A Tin WWT Nishijima R Yamakoshi K. 2021. Alteration of mucins in the submandibular gland during aging in mice. Arch Oral Biol. 121:104967. 33197804 10.1016/j.archoralbio.2020.104967

[bibr28-00220345211004842] Katsiougiannis S Wong DTW . 2016. The proteomics of saliva in Sjogren’s syndrome. Rheum Dis Clin North Am. 42(3):449–456. 27431347 10.1016/j.rdc.2016.03.004 PMC4955829

[bibr29-00220345211004842] Kronvall P Fahy TA Isaksson A Theander S Russell GFM . 1992. The clinical relevance of salivary amylase monitoring in bulimia-nervosa. Biol Psychiatry. 32(2):156–163. 1384726 10.1016/0006-3223(92)90018-u

[bibr30-00220345211004842] Mandel SJ Mandel L. 2003. Radioactive iodine and the salivary glands. Thyroid. 13(3):265–271. 12729475 10.1089/105072503321582060

[bibr31-00220345211004842] Mariette X Criswell LA . 2018. Primary Sjogren’s syndrome. N Engl J Med. 378(10):931–939. 29514034 10.1056/NEJMcp1702514

[bibr32-00220345211004842] Masters JM Noyce AJ Warner TT Giovannoni G Proctor GB . 2015. Elevated salivary protein in Parkinson’s disease and salivary DJ-1 as a potential marker of disease severity. Parkinsonism Relat Disord. 21(10):1251–1255. 26231472 10.1016/j.parkreldis.2015.07.021

[bibr33-00220345211004842] Miyagi Y Kondo Y Kusuda Y Hori Y Yamazaki S Munemasa T Mukaibo T Masaki C Hosokawa R. 2019. Submandibular gland-specific inflammaging-induced hyposalivation in the male senescence-accelerated mouse prone-1line (SAM-P1). Biogerontology. 20(4):421–432. 30684147 10.1007/s10522-019-09797-3

[bibr34-00220345211004842] Moreira Tdos S Takakura AC De Luca LA Jr Renzi A Menani JV . 2002. Inhibition of pilocarpine-induced salivation in rats by central noradrenaline. Arch Oral Biol. 47(6):429–434. 12102758 10.1016/s0003-9969(02)00031-6

[bibr35-00220345211004842] Nederfors T . 1996. Xerostomia: prevalence and pharmacotherapy. With special reference to beta-adrenoceptor antagonists. Swed Dent J Suppl. 116:1–70. 8813731

[bibr36-00220345211004842] Neyraud E Sayd T Morzel M Dransfield E. 2006. Proteomic analysis of human whole and parotid salivas following stimulation by different tastes. J Proteome Res. 5(9):2474–2480. 16944961 10.1021/pr060189z

[bibr37-00220345211004842] Osailan S Pramanik R Shirodaria S Challacombe SJ Proctor GB . 2011. Investigating the relationship between hyposalivation and mucosal wetness. Oral Dis. 17(1):109–114. 21029258 10.1111/j.1601-0825.2010.01715.x

[bibr38-00220345211004842] Osailan SM Proctor GB Carpenter GH Paterson KL McGurk M. 2006. Recovery of rat submandibular salivary gland function following removal of obstruction: a sialometrical and sialochemical study. Int J Exp Pathol. 87(6):411–423. 10.1111/j.1365-2613.2006.00500.x PMC2517394 17222209

[bibr39-00220345211004842] Pijpe J Kalk WWI Bootsma H Spijkervet FKL Kallenberg CGM Vissink A. 2007. Progression of salivary gland dysfunction in patients with Sjogren’s syndrome. Ann Rheum Dis. 66(1):107–112. 16728458 10.1136/ard.2006.052647 PMC1798390

[bibr40-00220345211004842] Proctor GB Asking B. 1989. A comparison between changes in rat parotid protein-composition 1 and 12 weeks following surgical sympathectomy. Q J Exp Physiol. 74(6):835–840. 2480619 10.1113/expphysiol.1989.sp003353

[bibr41-00220345211004842] Proctor GB Carpenter GH . 2007. Regulation of salivary gland function by autonomic nerves. Auton Neurosci. 133(1):3–18. 17157080 10.1016/j.autneu.2006.10.006

[bibr42-00220345211004842] Pushpass RAG Daly B Kelly C Proctor G Carpenter GH . 2019. Altered salivary flow, protein composition, and rheology following taste and TRP stimulation in older adults. Front Physiol. 10:652. 31214042 10.3389/fphys.2019.00652 PMC6555201

[bibr43-00220345211004842] Rathke H Kratochwil C Hohenberger R Giesel FL Bruchertseifer F Flechsig P Morgenstern A Hein M Plinkert P Haberkorn U , et al. 2018. Initial clinical experience performing sialendoscopy for salivary gland protection in patients undergoing ^225^Ac-PSMA-617 RLT. Eur J Nucl Med Mol Imaging. 46(1):139–147. 30151743 10.1007/s00259-018-4135-8

[bibr44-00220345211004842] Richards TM Hurley T Grove L Harrington KJ Carpenter GH Proctor GB Nutting CM . 2017. The effect of parotid gland-sparing intensity-modulated radiotherapy on salivary composition, flow rate and xerostomia measures. Oral Dis. 23(7):990–1000. 28434191 10.1111/odi.12686 PMC6157709

[bibr45-00220345211004842] Ruiz-Roca JA Pons-Fuster E Lopez-Jornet P. 2019. Effectiveness of the botulinum toxin for treating sialorrhea in patients with Parkinson’s disease: a systematic review. J Clin Med. 8(3):317. 10.3390/jcm8030317 PMC6463012 30845700

[bibr46-00220345211004842] Ryu OH Atkinson JC Hoehn GT Illei GG Hart TC . 2006. Identification of parotid salivary biomarkers in Sjogren’s syndrome by surface-enhanced laser desorption/ionization time-of-flight mass spectrometry and two-dimensional difference gel electrophoresis. Rheumatology. 45(9):1077–1086. 16522680 10.1093/rheumatology/kei212

[bibr47-00220345211004842] Scott J . 1977. Quantitative age-changes in histological structure of human submandibular salivary-glands. Arch Oral Biol. 22(3):221–227. 266877 10.1016/0003-9969(77)90158-3

[bibr48-00220345211004842] Scott J Gunn DL . 1991. A comparative quantitative histological investigation of atrophic changes in the major salivary-glands of liquid-fed rats. Arch Oral Biol. 36(11):855–857. 1763982 10.1016/0003-9969(91)90035-s

[bibr49-00220345211004842] Ship JA Decarli C Friedland RP Baum BJ . 1990. Diminished submandibular salivary flow in dementia of the Alzheimer type. J Gerontol. 45(2):M61–M66. 2313044 10.1093/geronj/45.2.m61

[bibr50-00220345211004842] Singer MC Marchal F Angelos P Bernet V Boucai L Buchholzer S Burkey B Eisele D Erkul E Faure F , et al. 2020. Salivary and lacrimal dysfunction after radioactive iodine for differentiated thyroid cancer: American Head and Neck Society Endocrine Surgery Section and Salivary Gland Section joint multidisciplinary clinical consensus statement of otolaryngology, ophthalmology, nuclear medicine and endocrinology. Head Neck. 42(11):3446–3459. 10.1002/hed.26417 32812307

[bibr51-00220345211004842] Tabak L Mandel ID Karlan D Baurmash H. 1978. Alterations in lactoferrin in salivary-gland disease. J Dent Res. 57(1):43–47. 277498 10.1177/00220345780570011801

[bibr52-00220345211004842] Takahashi S Takebuchi R Taniwaki H Domon T. 2018. Recovery of atrophic parotid glands in rats fed a liquid diet by switching to a pellet diet. Arch Oral Biol. 96:39–45. 30172944 10.1016/j.archoralbio.2018.08.015

[bibr53-00220345211004842] Takahashi S Uekita H Kato T Yuge F Ushijima N Inoue K Domon T. 2014. Immunohistochemical and ultrastructural investigation of acinar cells in submandibular and sublingual glands of rats fed a liquid diet. Tissue Cell. 46(2):136–143. 24553131 10.1016/j.tice.2014.01.001

[bibr54-00220345211004842] Tumilasci OR Cersosimo MG Belforte JE Micheli FE Benarroch EE Pazo JH . 2006. Quantitative study of salivary secretion in Parkinson’s disease. Mov Disord. 21(5):660–667. 16419045 10.1002/mds.20784

[bibr55-00220345211004842] Valdez IH Atkinson JC Ship JA Fox PC . 1992. Major salivary-gland function in patients with radiation-induced xerostomia - flow rates and sialochemistry. Int J Rad Oncol Biol Physics. 25(1):41–47. 10.1016/0360-3016(93)90143-j 8416881

[bibr56-00220345211004842] Valstar MH de Bakker BS de Steenbakkers RJHM Jong KH Smit LA Klein Nulent TJW van Es RJJ Hofland I de Keizer B Jasperse B , et al. 2020. The tubarial salivary glands: a potential new organ at risk for radiotherapy. Radiother Oncol [epub ahead of print 23 Sep 2020]. doi:10.1016/j.radonc.2020.09.034 32976871

[bibr57-00220345211004842] van Leeuwen SJM Potting CMJ Huysmans M Blijlevens NMA . 2019. Salivary changes before and after hematopoietic stem cell transplantation: a systematic review. Biol Blood Marrow Transplant. 25(6):1055–1061. 10.1016/j.bbmt.2019.01.026 30710684

[bibr58-00220345211004842] van Leeuwen SJM Proctor GB Potting CMJ ten Hoopen S van Groningen LFJ Bronkhorst EM Blijlevens NMA Huysmans M. 2018. Early salivary changes in multiple myeloma patients undergoing autologous HSCT. Oral Dis. 24(6):972–982. 29637662 10.1111/odi.12866

[bibr59-00220345211004842] Veerman ECI vandenKeybus PAM Vissink A Amerongen AVN . 1996. Human glandular salivas: their separate collection and analysis. Eur J Oral Sci. 104(4):346–352. 10.1111/j.1600-0722.1996.tb00090.x 8930581

[bibr60-00220345211004842] Vissink A Mitchell JB Baum BJ Limesand KH Jensen SB Fox PC Elting LS Langendijk JA Coppes RP Reyland ME . 2010. Clinical management of salivary gland hypofunction and xerostomia in head-and-neck cancer patients: successes and barriers. Int J Radiat Oncol Biol Phys. 78(4):983–991. 10.1016/j.ijrobp.2010.06.052 PMC2964345 20970030

[bibr61-00220345211004842] Wang Z Shen MM Liu XJ Si Y Yu GY . 2015. Characteristics of the saliva flow rates of minor salivary glands in healthy people. Arch Oral Biol. 60(3):385–392. 25526622 10.1016/j.archoralbio.2014.11.016

[bibr62-00220345211004842] Wolff A Joshi RK Ekstrom J Aframian D Pedersen AML Proctor G Narayana N Villa A Sia YW Aliko A , et al. 2017. A guide to medications inducing salivary gland dysfunction, xerostomia, and subjective sialorrhea: a systematic review sponsored by the world workshop on oral medicine VI. Drugs R D. 17(1):1–28. 27853957 10.1007/s40268-016-0153-9 PMC5318321

[bibr63-00220345211004842] Zhang YQ Ye X Meng Y Zhao YN Liu DG Yu GY . 2019. Evaluation of parotid gland function before and after endoscopy-assisted stone removal. J Oral Maxillofac Surg. 77(2):328.e1–328.e9. 10.1016/j.joms.2018.09.034 30395822

