# Supplementary material for: Disease-Induced Changes in Salivary Gland Function and the Composition of Saliva
Source: J Dent Res. 2021 Apr 17;100(11):1201–9. doi: 10.1177/00220345211004842 (PMC8461045; doi:10.1177/00220345211004842)
Supplement: sj-pdf-1-jdr-10.1177_00220345211004842 – Supplemental material for Disease-Induced Changes in Salivary Gland Function and the Composition of Saliva [file sj-pdf-1-jdr-10.1177_00220345211004842.pdf]

Disease Induced Changes in Salivary Gland Function and the Composition of Saliva

Appendix

Salivary gland secretion.

Stimulation-secretion coupling. Activation of m3AChRs and to a lesser extent m1AChRs in some salivary glands (Tobin et al. 2009) by acetylcholine released from parasympathetic secretomotor nerves provides the principal drive for acinar cell secretion of electrolyte and water into the lumina of acini. Signalling in acinar cells is via inositol triphosphate (IP_3) and raised cytoplasmic Ca^{2+} concentration. Noradrenaline release from sympathetic nerves similarly activates small amounts of fluid secretion through acinar cell α_1-adrenoceptors. Noradrenaline activation of β_1-adrenoceptors provides a strong drive for protein secretion in serous acinar cells through raised intracellular cAMP and activation of exocytosis through protein kinase A (PKA). Neuropeptide co-transmitters provide additional drive for secretion, for example vasoactive intestinal polypeptide (VIP) released from parasympathetic nerves evokes protein secretion through VIP (VIPAC) receptors coupled through intracellular cAMP. Secretion of mucin by acinar cells of mucous glands, as demonstrated in the rat sublingual gland, is evoked by parasympathetic nerve mediated stimuli and release of acetylcholine acting through m3AChRs and m1AChRs and generation of intracellular diacylglycerol (DAG) and protein kinase C (PKC) activity. VIP also stimulates mucin secretion, although to a lesser extent than muscarinic receptor activation, via VIPAC receptors and cAMP coupling, without involvement of sympathetic nerve signalling (Culp et al. 2020). Protein secretion is augmented by crosstalk between the Ca^{2+} and cAMP intracellular coupling pathways in serous and mucous acinar cells (Figure 2a) (Culp et al. 2020; Proctor and Carpenter 2007).

Fluid secretion. The initiation of electrolyte secretion into acinar lumina is via an apical Cl^- channel (Tmem16a) that opens when intracellular Ca^{2+} is increased. Paracellular movement of Na^+ through leaky tight junctions follows Cl^- into lumina and the accumulating salt concentration drives paracellular and some transcellular

movement of water leading to formation of an isotonic saliva in the acinar lumen. Saliva then enters the ductal system where (striated) duct cell membrane transport proteins modify the composition of saliva, most notably by secreting K^+ and HCO_3^- (generated by the action of intracellular carbonic anhydrase 2) and reabsorbing Na^+ and Cl^-. The duct-modified saliva secreted into the mouth is hypotonic, a feature that facilitates detection of dietary salt in the mouth. On reflex stimulation of secretion during feeding, salivary HCO_3^- and Na^+ concentrations are higher than during resting (unstimulated) salivary secretion (Figure 2b, main text); (Catalan et al. 2014; Lee et al. 2012).

Protein secretion. Most of the protein present in saliva is actively secreted by acinar cells through regulated exocytosis (Castle and Castle 1998). In general, the concentrations of salivary proteins that are actively transported, for example amylase, mucins, statherins, proline-rich proteins and carbonic anhydrase 6 are maintained as the flow of saliva increases (Oppenheim et al. 2007), and these proteins can be considered to form a core salivary proteome, largely responsible for creating the properties and fulfilling the core functions of saliva (Ruhl 2012). The fusion of protein storage granules with the apical membrane has been observed in real time in green fluorescent protein labelled mice and such experiments have revealed the role of actin filament rearrangement in enabling granule fusion in parotid acinar cells (Messenger et al. 2014; Tran et al. 2015). Other secretory pathways that make a lesser contribution to protein secretion are present in acinar cells. A constitutive pathway traffics small vesicles directly to the plasma membrane from the trans-golgi network and a constitutive-like pathway involves direct secretion from immature protein storage granules by-passing a maturation phase (Huang et al. 2001; Proctor et al. 2003). Transcytosis of dimeric IgA also involves a vesicular pathway (Johansen et al. 2001). Glandular saliva can also contain low amounts of proteins and macromolecules (<40kD mw) that have diffused across leaky acinar cell tight junctions as demonstrated following salivary gland stimulation with adrenergic secretagogues (Segawa 1994). Some proteins are secreted by ductal cells, including lactoferrin, lysozyme, tissue kallikreins and protein growth factors, all of which have been localized in ductal cells in humans or animal models (Garrett and Anderson 1991; Garrett et al. 1991; Gresik 1994; Reitamo et al. 1980; StanevaDobrovski 1997).

References

Castle D, Castle A. 1998. Intracellular transport and secretion of salivary proteins. Critical Reviews in Oral Biology & Medicine. 9(1):4-22.

Catalan MA, Pena-Munzenmayer G, Melvin JE. 2014. Ca2+-dependent k+ channels in exocrine salivary glands. Cell Calcium. 55(6):362-368.

Culp DJ, Zhang Z, Evans RL. 2020. Vip and muscarinic synergistic mucin secretion by salivary mucous cells is mediated by enhanced pkc activity via vip-induced release of an intracellular ca2+ pool. Pflugers Archiv-European Journal of Physiology. 472(3):385-403.

Garrett JR, Anderson LC. 1991. Rat sublingual salivary-glands - secretory changes on parasympathetic or sympathetic-nerve stimulation and a reappraisal of the adrenergic-innervation of striated ducts. Archives of Oral Biology. 36(9):675-683.

Garrett JR, Suleiman AM, Anderson LC, Proctor GB. 1991. Secretory responses in granular ducts and acini of submandibular glands in vivo to parasympathetic or sympathetic nerve stimulation in rats. Cell and Tissue Research. 264(1):117-126.

Gresik EW. 1994. The granular convoluted tubule (gct) cell of rodent submandibular glands. Microscopy Research and Technique. 27(1):1-24.

Huang AY, Castle AM, Hinton BT, Castle JD. 2001. Resting (basal) secretion of proteins is provided by the minor regulated and constitutive-like pathways and not granule exocytosis in parotid acinar cells. Journal of Biological Chemistry. 276(25):22296-22306.

Johansen FE, Braathen R, Brandtzaeg P. 2001. The j chain is essential for polymeric ig receptor-mediated epithelial transport of iga. Journal of Immunology. 167(9):5185-5192.

Lee MG, Ohana E, Park HW, Yang D, Muallem S. 2012. Molecular mechanism of pancreatic and salivary gland fluid and hco3 secretion. Physiol Rev. 92(1):39-74.

Messenger SW, Falkowski MA, Groblewski GE. 2014. Ca2+-regulated secretory granule exocytosis in pancreatic and parotid acinar cells. Cell Calcium. 55(6):369-375.

Oppenheim FG, Salih E, Siqueira WL, Zhang WM, Helmerhorst EJ. 2007. Salivary proteome and its genetic polymorphisms. Oral-Based Diagnostics. 1098:22-50.

Proctor GB, Carpenter GH. 2007. Regulation of salivary gland function by autonomic nerves. Autonomic Neuroscience-Basic & Clinical. 133(1):3-18.

Proctor GB, Carpenter GH, Segawa A, Garrett JR, Ebersole L. 2003. Constitutive secretion of immunoglobulin a and other proteins into lumina of unstimulated submandibular glands in anaesthetised rats. Exp Physiol. 88(1):7-12.

Reitamo S, Konttinen YT, Segerbergkonttinen M. 1980. Distribution of lactoferrin in human salivary-glands. Histochemistry. 66(3):285-291.

Ruhl S. 2012. The scientific exploration of saliva in the post-proteomic era: From database back to basic function. Expert Review of Proteomics. 9(1):85-96.

Segawa A. 1994. Tight junctional permeability in living cells - dynamic changes directly visualized by confocal laser microscopy. Journal of Electron Microscopy. 43(5):290-298.

StanevaDobrovski L. 1997. Lysozyme expression in the rat parotid gland: Light and electron microscopic immunogold studies. Histochemistry and Cell Biology. 107(5):371-381.

Tobin G, Giglio D, Lundgren O. 2009. Muscarinic receptor subtypes in the alimentary tract. Journal of Physiology and Pharmacology. 60(1):3-21.

Tran DT, Masedunskas A, Weigert R, Ten Hagen KG. 2015. Arp2/3-mediated f-actin formation controls regulated exocytosis in vivo. Nature Communications. 6.
